# Pharmaceutical iron formulations do not cross a model of the human blood-brain barrier

**DOI:** 10.1371/journal.pone.0198775

**Published:** 2018-06-11

**Authors:** Brian Chiou, Emma H. Neal, Aaron B. Bowman, Ethan S. Lippmann, Ian A. Simpson, James R. Connor

**Affiliations:** 1 Department of Neurosurgery, Penn State Hershey Medical Center, Hershey, PA, United States of America; 2 Department of Chemical and Biomolecular Engineering, Vanderbilt University, Nashville, TN, United States of America; 3 Department of Pediatrics, Vanderbilt University Medical Center, Nashville, TN, United States of America; 4 Department of Neurology, Vanderbilt University Medical Center, Nashville, TN, United States of America; 5 Department of Biochemistry, Vanderbilt University Medical Center, Nashville, TN, United States of America; 6 Department of Biomedical Engineering, Vanderbilt University, Nashville, TN, United States of America; 7 Department of Neural and Behavioral Sciences, Penn State Hershey Medical Center, Hershey, PA, United States of America; Hungarian Academy of Sciences, HUNGARY

## Abstract

Whether iron formulations used therapeutically for a variety of conditions involving iron deficiency can deliver iron to the brain is a significant clinical question given the impact that iron loading has on the brain in neurodegenerative diseases. In this study, we examine the ability of 5 pharmaceutical iron formulations that are given intravenously for treatment of iron deficiency to cross an *in vitro* model of the blood-brain barrier. The model uses human brain endothelial cells derived from induced pluripotent stem cells. We report that, compared to the natural iron delivery proteins, transferrin and H-ferritin, the pharmaceutical iron formulations neither cross the blood-brain barrier model nor significantly load the endothelial cells with iron. Furthermore, we report that mimicking brain iron sufficiency or deficiency by exposing the endothelial cells to apo- or holo-transferrin does not alter the amount of iron compound transported by or loaded into the cells. Coupled with previous studies, we propose that pharmaceutical iron formulations must first be processed in macrophages to make iron bioavailable. The results of this study have significant clinical and mechanistic implications for the use of therapeutic iron formulations.

## Introduction

Iron is a crucial micronutrient serving as a cofactor in various cellular processes such as myelination, oxygen transport, and DNA synthesis [1]. However, as a transition element, it has properties enabling generation of oxygen free radicals and oxidative stress through the Fenton reaction [2]. As a result, iron levels are tightly regulated because both too much iron as well as a deficiency in iron can be detrimental to biological function and health [3–7].

Iron deficiency (ID) is the most common and widespread nutritional disorder with over 2 billion people suffering significant negative health effects worldwide [8]. In children, ID impedes mental and motor development leading to lifelong muscular and cognitive deficiencies [9–11]. In adults, ID leads to extreme fatigue, reduced work capacity and physical performance, hearing loss, recurrent infection, heart failure morbidity, and general reduced quality of life [8,12,13]. There is a widespread, serious misperception that oral iron supplements are safe and effective at alleviating ID. In a recent Cochrane review of 61 clinical trials, women taking oral iron supplements had just a 38% decreased risk of ID at the end of treatment compared to placebo [14].

Intravenous iron delivery is an option for iron supplementation, particularly in persons with conditions such as heavy uterine bleeding or anemia of chronic disease in which oral iron uptake from the gut may be limited due to inflammation. Although intravenous infusion of iron may present a potentially more effective method of iron supplementation [15], concerns exist regarding the safety of intravenous iron delivery [16–19]. One concern is how repeated iron supplementation may impact brain iron load. Although uptake of iron from the blood into the brain is subject to regulation by the blood-brain barrier (BBB), excess iron in the brain is associated with Parkinson’s and Alzheimer’s disease, amyotrophic lateral sclerosis, and neurodegeneration with brain iron accumulation [20]. Conversely, intravenous iron treatment is under clinical investigation for use in neurological syndromes such as Restless Legs syndrome [21].

Our recent studies have shown that brain iron transport via transferrin is not a simple transcytotic process as once taught [22]. A direct transcytosis model does not account for regulation of iron uptake into the brain nor does it account for the iron requirements of the metabolically active endothelial cells. Thus we proposed a data-based model that reveals iron is released into the cytoplasm of the endothelial cells where it can be stored in ferritin if not immediately used. Moreover, iron can be released from the endothelial cells in response to the ratio of holo- (iron loaded) to apo- (iron poor) transferrin on the brain side of the BBB [22,23]. Therefore, the question of transport of iron into the brain by different chemical formulations must begin with investigation of the potential for transport of iron across the BBB. This study is the first to directly interrogate the ability of various intravenous pharmaceutical iron formulations that are commonly used in the clinic to treat systemic iron deficiency, including Feraheme (ferumoxytol), Venofer (iron sucrose), Dexferrum (iron dextran), Injectafer (ferric carboxymaltose), and Ferrlecit (sodium ferric gluconate) to cross the BBB.

## Materials and methods

### Human brain endothelial cell culture

Human brain endothelial cells (huECs) were differentiated from CC3 induced pluripotent stem cell (iPSC) lines as previously described [24–28]. Briefly, iPSCs were maintained in E8 medium (prepared in-house) [29] on growth factor reduced Matrigel (Corning) and passaged using Versene (Thermo Fisher Scientific) upon reaching approximately 70% confluence. For differentiation, cells were washed once with DPBS (Thermo Fisher Scientific) and incubated with Accutase (Stem Cell Technologies) for 3 min at 37°C to yield a single cell suspension, followed by collection via centrifugation. Cell density and viability were measured using a Countess II (Thermo Fisher Scientific) and Trypan Blue stain (Thermo Fisher Scientific), and cells were plated at a density of 15,600 live cells per square centimeter in E8 medium supplemented with 10 μM Rho-associated, coiled-coil containing protein kinase (ROCK) inhibitor Y27632 (R&D Systems). Approximately 24 h after seeding, differentiation was initiated by media change to E6 media (D0) [30]. E6 media was changed every 24 h for 4 days. On day 4 of differentiation, media was changed to human endothelial serum-free media (hESFM, Thermo Fisher Scientific) plus 1% platelet poor platelet derived serum (PDS, Alfa Aesar), collectively referred to as EC medium, supplemented with 20 ng/mL basic fibroblast growth factor (bFGF, Peprotech) and 10 μM all-trans retinoic acid (RA, Sigma). Media was not changed for 48 h. On day 6 of differentiation, cells were washed once with DPBS and incubated with Accutase at 37°C until dissociated into an approximately single cell suspension. Cells were collected via centrifugation and resuspended in freeze medium consisting of 60% EC medium containing 20 ng/mL bFGF, 30% fetal bovine serum (Thermo Fisher Scientific), and 10% Hybri-Max DMSO (Sigma), supplemented with 10 μM Y27632 and 10 μM RA [31]. Cells were frozen overnight at -80°C in an isopropanol-filled freezing container (Thermo Fisher Scientific) before being transferred to long-term storage in liquid nitrogen.

Transwell filters (24-well Costar Transwell, 0.4 μm pore, polyethylene terephthalate, Corning) were coated with a mixture of collagen IV (1 mg/mL, Sigma) and fibronectin (1 mg/mL, Sigma) at a ratio of 5 parts UltraPure H_2_O (Thermo Fisher Scientific), 4 parts Collagen IV, and 1 part Fibronectin. A total of 150 μL of this mixture was used to coat the filters overnight at 37°C. Cryo-preserved huECs were thawed and then plated overnight at a density of 25,000 huECs per Transwell filter in the apical chamber in 150 μL of EC medium supplemented with 10 μM Y27632, 10 μM RA, and 20 ng/mL bFGF. The basal chamber was filled with 600 μL of the same media. The next day, media was changed in both the apical and basal chamber to EC medium supplemented with 10 μM Y27632 but lacking bFGF and RA. The following day, all experiments were performed. At each step, cells were incubated at 37°C with 5% CO_2_.

### Protein preparation

Recombinant H-ferritin was prepared as previously described [32]. Briefly, wild-type human H-ferritin containing a poly-His tag was subcloned into the pET30a(+) vector, to be produced in BL21 *Escherichia coli*. Isopropyl-β-D-thio-galactoside (IPTG, Sigma) was used to induce expression. Following induction, bacteria were lysed in a mixture of Bugbuster (Novagen), benzonase nuclease (VWR), bacterial protease inhibitor cocktail (Sigma) and lysozyme (Roche). H-ferritin protein was purified using a nickel column (GE Healthcare Bio-Sciences) according to the manufacturer’s instructions. Identity of H-ferritin was verified by western blot (*data not shown*).

Apo-transferrin (apo-Tf, Sigma) and H-ferritin were iron loaded with ferric chloride hexahydrate (FeCl_3_, Sigma), nitrilotriacetic acid (NTA, Sigma), and sodium bicarbonate complexed at a ratio of 100 μL NTA: 13.4 μL FeCl_3_: 23.3 μL NaHCO_3_. This solution was allowed to complex for 30 minutes to create the Fe-NTA complex. Apo-Tf and H-ferritin were incubated with the Fe-NTA complex for 30 additional minutes to allow for sufficient iron loading [22,23].

### Transport studies

Prior to the start of the experiment, both the apical and basal chambers were washed with 1X PBS and underwent a complete media exchange into hESFM plus 10 μM Y27632, to remove serum. After media addition, Trans-Endothelial Electrical Resistance (TEER) readings were taken using an Epithelial Volt/Ohm Meter for TEER (EVOM2 with STX2 electrodes, World Precision Instruments) [33]. Blank (media only) TEER values were subtracted from all other TEER measurements. 4.4 kDa TRITC-Dextran (Sigma) was added to the apical chamber at the start of each experiment with the experimental treatment and assayed from the basal chamber (Excitation: 557 nm, Emission: 576 nm) at each timepoint to assess tight junction formation and barrier permeability using a SpectraMax Gemini EM plate reader (Molecular Devices).

At the start of the experiment, 300 μg/mL of each intravenous iron formulation, holo-transferrin (holo-Tf), or H-ferritin was added to the apical chamber. This concentration of iron formulations was chosen as the most clinically relevant concentration and was also informed by our previous cytotoxicity studies in macrophages [34]. Furthermore, 1 mg/mL apo-Tf or holo-Tf was added to the basal chamber where indicated, doses we have previously used in previous studies by our lab [34]. At 2, 4, 6, 8 and 24 hours, samples were taken from the basal chamber and assayed for TRITC (as a control for any leakage of the tight junctions) signal and iron content. At 24 hours, the endothelial cells were harvested and analyzed for iron content. For all cases, blank (no treatment) controls were subtracted from all other measurements.

Samples (100 μL) at each time point were analyzed for iron content via inductively coupled plasma mass spectrometry (ICP-MS) with Collision Cell Technology (CCT). Prior to analysis, samples were digested in 8 N nitric acid at 60°C overnight. This solution was diluted to a final concentration of 0.3 N nitric acid and analyzed via ICP-MS (Thermo Fisher Scientific X Series 2). Samples were analyzed by an external calibration method using standards with concentrations ranging between 0 and 100 parts per billion (ppb). Reported measurements of iron in each sample represent the average of 3 measurements.

### Statistical analysis

Statistical analysis was performed using Graphpad Prism 4 software (GraphPad Software, Inc.). Data from three technical replicates were averaged and are expressed as the mean ± standard deviation (SD). Two-way ANOVA with Bonferroni *post-hoc* analysis was used where appropriate. A p-value of <0.05 was considered significant.

## Results

**Fig 1:**
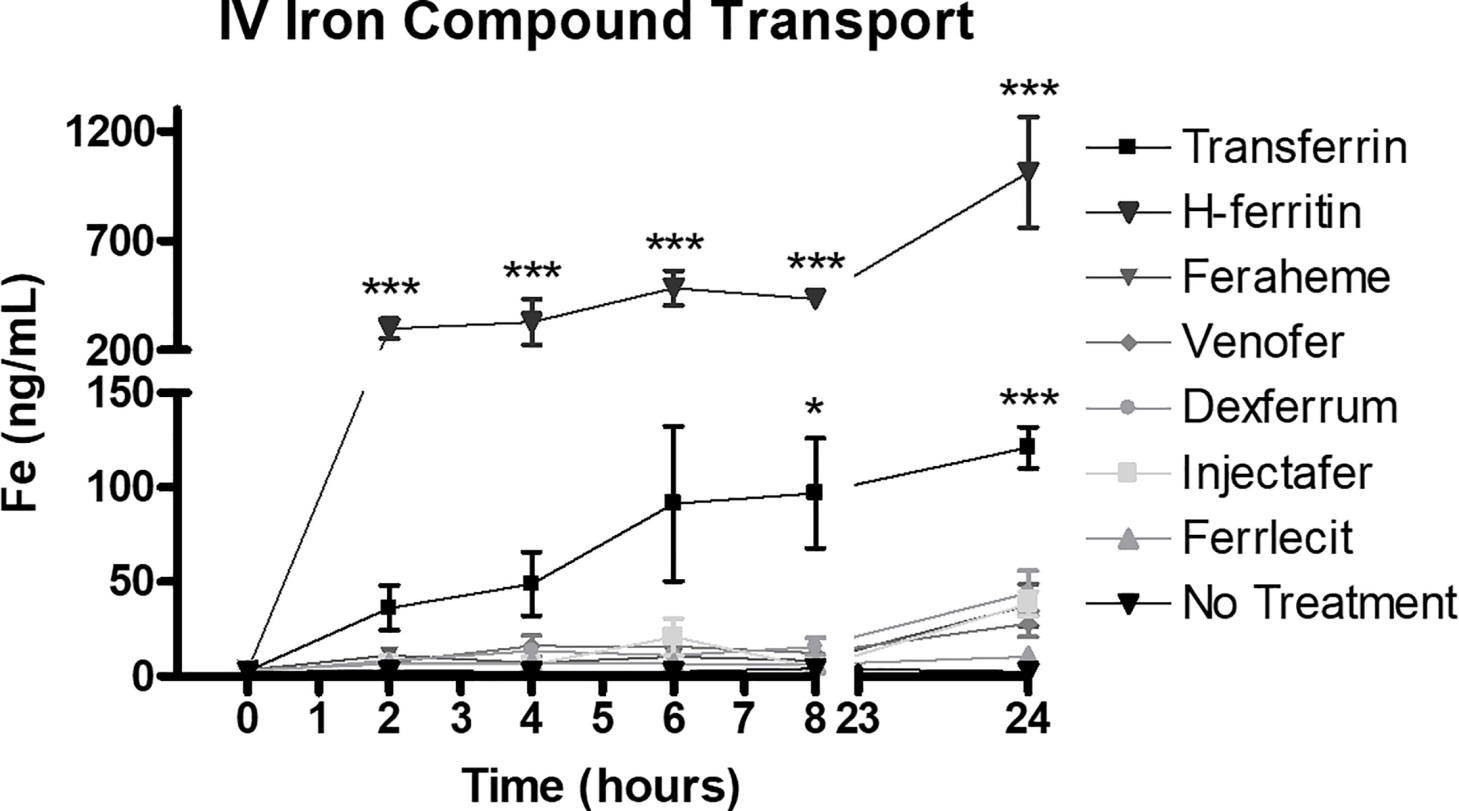
Commercial iron formulations do not transport iron across a BBB model. Using the bichamber model of the BBB, H-ferritin and Tf robustly transport iron across the huECs at each time point. Minimal iron transport is observed for the 5 commercial iron formulations. The concentration of the commercial formulations, Tf and H-ferritin was 300 μg/ml. All values are means ± SD, with statistical significance evaluated against the untreated control using two-way ANOVA and Bonferroni’s *post hoc* comparisons. * = p<0.05, *** = p<0.001.

Using an *in vitro* model of the BBB, we first examined the ability of transferrin, H-ferritin, or the iron formulations to promote iron transport across the huECs. Transferrin and H-ferritin were included in the experiment as positive controls because previous studies have shown transport of both of these proteins [22,23,35], while untreated cells were used to establish baseline measurements (Fig 1). A 4.4 kDa TRITC-Dextran complex was included as a readout for barrier permeability; <0.5% leakage was observed. Furthermore, TEER values from this experiment averaged 1939 ± 314 Ohms x cm^2^ across all wells. These values are much greater than our previous studies using primary cells and in line with previous studies with these cells [22,23,27]. The total iron transport at 24 hours for each of five iron formulations represented less than 0.02% of the total iron formulation (Fig 1). Of the five compounds, Dexferrum was associated with the highest amount of iron observed in the basal chamber (44.13 ng/mL); but this represented only 0.015% of the total Dexferrum initially added to the apical chamber. In comparison, transferrin (127.71 ng/mL) and H-ferritin (1011.9 ng/mL) transported significantly more iron across the huECs at 24 hours (p<0.001), representing 9% and 10% of the total exposure respectively.

**Fig 2:**
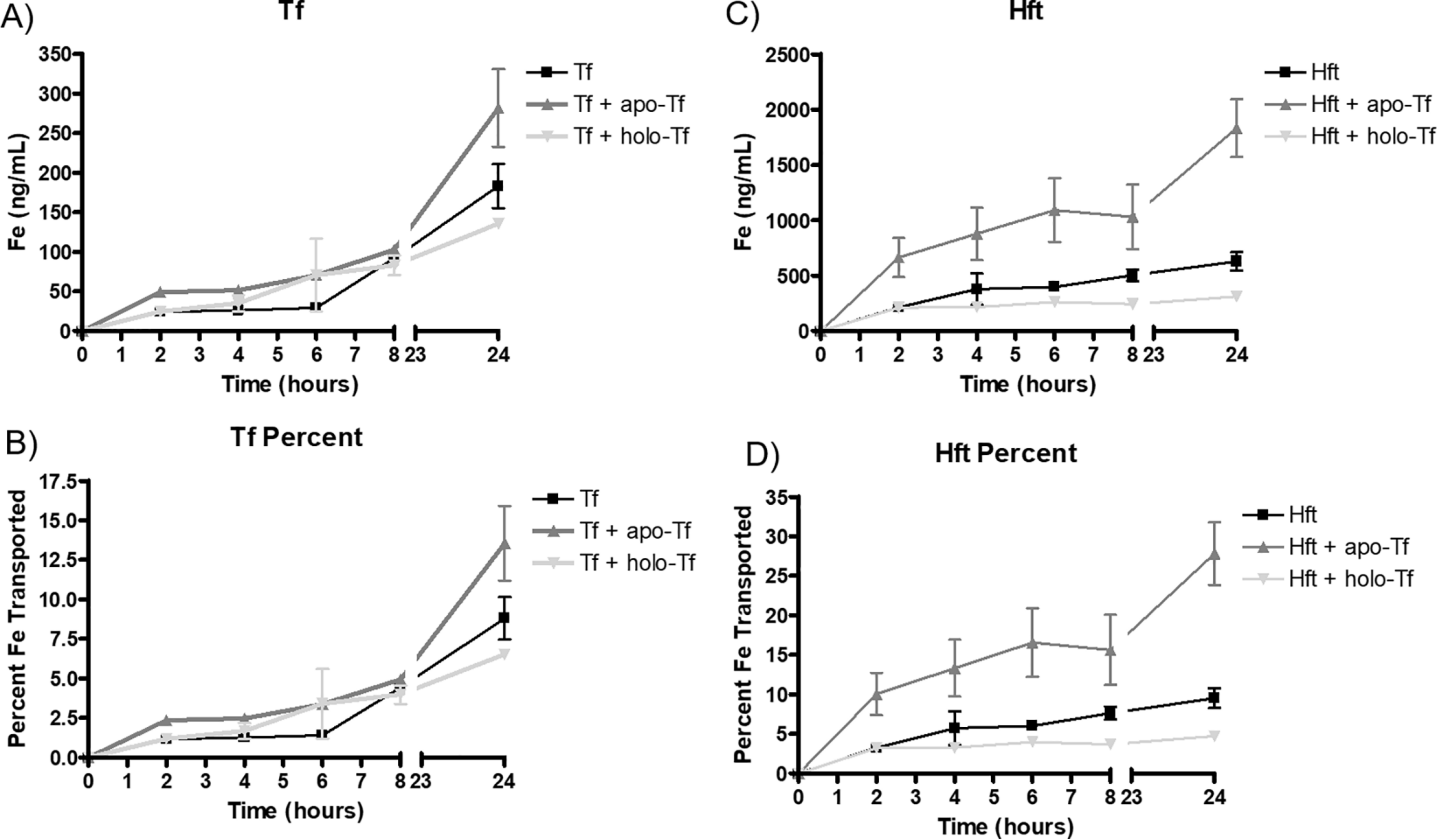
Transferrin and H-ferritin transport of iron is responsive to signaling by apo- and holo-Tf. **A)** Apo-Tf in the basal chamber increases the amount of iron transported across the endothelial cells by transferrin whereas holo-Tf decreases the amount transported. The control condition is the basal media with no extra addition. **B)** Here, the data from panel A are shown as a percent of control. **C)** Apo-Tf in the basal chamber significantly increases the amount of iron transported by H-ferritin while holo-Tf decreases the amount transported relative to control. **D)** H-ferritin transport of iron from panel C as a percent of control conditions. All values are means ± SD, with statistical significance evaluated against the control (addition of Tf or Hft alone) using two-way ANOVA and Bonferroni’s *post hoc* comparisons. * = p<0.05, *** = p<0.001.

We have previously established that the iron status of Tf in the basal chamber of the BBB model can signal to the endothelial cells and significantly influence the amount of iron that is transported across or released from the BBB model [22,23]. We added either 1 mg/mL apo-Tf or holo-Tf to the basal chamber of the Transwell bichamber model at the start of the experiment. Apo-Tf significantly increased the amount of iron transported from both transferrin and H-ferritin whereas the addition of holo-Tf decreased the total amount of iron transported for both proteins (Fig 2). In the case of transferrin, the highest amount of iron transported at 24 hours was in the apo-Tf condition (281.9 ± 84.9 ng/mL), while H-ferritin + apo-Tf in the basal chamber (1833.0 ± 451.2 ng/mL) represented the highest overall transport of iron across all conditions (Table 1). There was no significant change in the amount of iron transported for any of the iron formulations in the presence of either apo- or holo-Tf in the basal chamber. Moreover, even with the addition of apo-Tf or holo-Tf, total percent iron transport still did not exceed 0.02% of the initial addition (Table 1).

**Table 1:** Transport of iron across the BBB.

	Time (hours)				
Treatment	2	4	6	8	24
	Iron Values Reported as ng/mL				
**Transferrin**	24.2 ± 2.0	26.2 ± 1.7	29.2 ± 8.6	92.0 ± 8.5	183.2 ± 48.3
**Tf + apo-Tf**	49.1 ± 3.9	51.4 ± 5.9	70.3 ± 10.1	102.7 ± 11.8	281.9 ± 84.9**
**Tf + holo-Tf**	24.7 ± 0.9	35.2 ± 17.6	70.7 ± 19.2	82.9 ± 21.3	135.7 ± 11.4
**H-Ferritin**	214.1 ± 10.4	377.7 ± 24.4	397.5 ± 37.0	503.5 ± 91.0	628.1 ± 144.2
**Hft + apo-Tf**	662.8 ± 204.4	877.2 ± 309.3	1091.1 ± 296.6	1030.4 ± 305.1	1833.0 ± 451.2**
**Hft + holo-Tf**	212.0 ± 12.9	215.4 ± 19.2	261.9 ± 22.7	242.3 ± 12.9	311.9 ± 29.5
**Feraheme**	9.4 ± 0.2	12.0 ± 1.0	8.4 ± 0.3	11.1 ± 3.3	8.4 ± 0.5
**Feraheme + apo-Tf**	9.2 ± 0.7	15.9 ± 0.2	11.3 ± 5.2	22.7 ± 2.1	21.4 ± 15.9
**Feraheme + holo-Tf**	11.5 ± 2.7	17.9 ± 4.5	12.3 ± 5.6	18.2 ± 3.6	9.2 ± 2.0
**Venofer**	9.9 ± 2.7	18.3 ± 9.7	13.2 ± 3.4	20.1 ± 8.4	13.7 ± 4.7
**Venofer + apo-Tf**	7.8 ± 5.2	9.2 ± 1.6	4.8 ± 0.3	8.5 ± 1.7	6.2 ± 0.1
**Venofer + holo-Tf**	15.5 ± 2.5	17.6 ± 3.4	17.9 ± 3.2	20.8 ± 7.3	18.9 ± 5.0
**Dexferrum**	13.1 ± 3.5	19.7 ± 8.1	19.0 ± 6.6	33.5 ± 19.6	46.1 ± 22.1
**Dexferrum + apo-Tf**	20.5 ± 14.4	23.1 ± 9.3	20.2 ± 4.2	19.8 ± 7.9	22.2 ± 4.6
**Dexferrum + holo-Tf**	10.1 ± 1.9	11.3 ± 4.8	16.1 ± 3.1	15.8 ± 8.2	19.1 ± 6.6
**Injectafer**	8.5 ± 2.0	5.0 ± 0.6	19.8 ± 9.4	8.5 ± 4.1	34.9 ± 10.4
**Injectafer + apo-Tf**	12.5 ± 4.4	12.1 ± 4.3	19.8 ± 9.0	11.8 ± 3.8	47.5 ± 20.0
**Injectafer + holo-Tf**	8.4 ± 6.8	13.6 ± 11.9	5.5 ± 0.4	20.1 ± 2.2	5.9 ± 1.0
**Ferrlecit**	13.5 ± 2.2	19.4 ± 4.3	16.4 ± 2.3	13.6 ± 2.4	27.7 ± 17.8
**Ferrlecit + apo-Tf**	5.3 ± 0.6	6.3 ± 1.0	5.0 ± 0.4	9.6 ± 1.7	27.3 ± 2.3
**Ferrlecit + holo-Tf**	11.3 ± 7.9	16.5 ± 2.7	15.0 ± 5.2	13.7 ± 1.1	16.2 ± 10.7

Presented here are the raw values of iron transport in ng/mL at times 2, 4, 6, 8, and 24 hours post addition of iron formulations to the apical chamber. Apo-Tf significantly increased the amount of Tf and Hft bound iron transported, but did not affect transport of the iron formulations. All values are means ± SD, statistical significance evaluated against the control (addition of Tf or Hft alone at 24 hours) using two-way ANOVA and Bonferroni’s *post hoc* comparisons.

** = p<0.01.

We also found that the iron formulations do not load the huECs with iron. We compared the iron loading of huECs by the 5 IV iron formulations, Tf, and Hft across three conditions: a control condition that had no Tf added to the basal chamber and two experimental conditions where 1 mg/mL apo-Tf or holo-Tf was added to the basal chamber media. The addition of apo- or holo-Tf to the basal media failed to significantly increase iron loading of the cells by the iron formulations (Table 2). In contrast, both Tf and Hft significantly increased the iron content of the huECs over control. Moreover, the addition of apo-Tf to the basal chamber increased the iron content of the huECs by 50.4% for iron loading by Tf and 87.6% for Hft whereas the presence of holo-Tf in the basal chamber decreased iron loading by Tf by 22.9% and 54.0% by Hft (p<0.001 for all values). In these experiments, the total amount of TRITC-Dextran leakage observed was <0.5%. The TEER values across all conditions averaged 1728 ± 424 Ohms x cm^2^.

**Table 2:** Iron loading of endothelial cells.

	Control (ng/mL)	+apo-Tf (ng/mL)	+holo-Tf (ng/mL)
**Transferrin**	580.9 ± 121.7	874.0 ± 49.5***	448.1 ± 11.8***
**H-ferritin**	1176.9 ± 304.1	2207.3 ± 170.6***	541.6 ± 87.3***
**Feraheme**	22.0 ± 2.7	24.8 ± 3.7	6.3 ± 0.2
**Venofer**	92.9 ± 11.2	19.4 ± 2.1	98.9 ± 1.8
**Dexferrum**	31.0 ± 10.6	29.1 ± 5.0	22.5 ± 5.8
**Injectafer**	31.5 ± 3.2	30.0 ± 4.9	41.0 ± 4.2
**Ferrlecit**	75.3 ± 2.2	75.1 ± 9.3	65.9 ± 13.8
**No Treatment**	5.9 ± 0.8	7.2 ± 2.5	5.2 ± 0.7

After 24 hours of exposure to the different iron formulations, endothelial cells were harvested and their iron content determined by ICP-MS, presented here as ng/mL. There was iron loading by the different formulations compared to control (no treatment). However, this iron loading was much less compared to loading by Tf or Hft. All values are means ± SD, statistical significance evaluated against the control (addition of Tf or Hft alone) using two-way ANOVA and Bonferroni’s *post hoc* comparisons.

*** = p<0.001.

## Discussion

The development of therapeutic iron compounds that can be given intravenously represents a potentially effective and rapid method for the treatment of iron deficiency. The results of this study demonstrate that there is no inherent ability of these pharmaceutical iron formulations to be taken up by the human brain endothelial cells or for the iron to be transported across the cells and released. The addition of a stimulus such as apo-Tf, which has been shown in our model to increase iron transport and release [22,23] and confirmed in this study to occur in human brain endothelial cells, had no impact on the iron formulations. The ability of apo-Tf and holo-Tf to alter iron transport and release has been posited by our group to represent signaling regarding brain iron status and to account for local iron regulation in the brain [23].

In this study, we used human brain endothelial cells that have been differentiated from iPSCs [26] to examine the ability of 5 iron formulations to be transported in a novel *in vitro* BBB model. Transferrin and H-ferritin are established as natural iron delivery proteins and thus served as positive controls [33]. Here, we demonstrate that the addition of the various iron formulations resulted in less than 0.02% iron transport across the BBB, even in the presence of apo-Tf. In comparison, both transferrin and H-ferritin robustly transported iron. Furthermore, we demonstrate that 24 hour exposure to these pharmaceutical iron formulations does not result in iron loading of the human endothelial cells. Tthe corresponding exposure to Hft or Tf for the same time resulted in significant iron loading. These results are consistent with our reports on bovine microvasculature BBB models [23] and microvasculature from human brain that endothelial cells of the BBB load iron and can serve as an iron reservoir [36].

**Fig 3:**
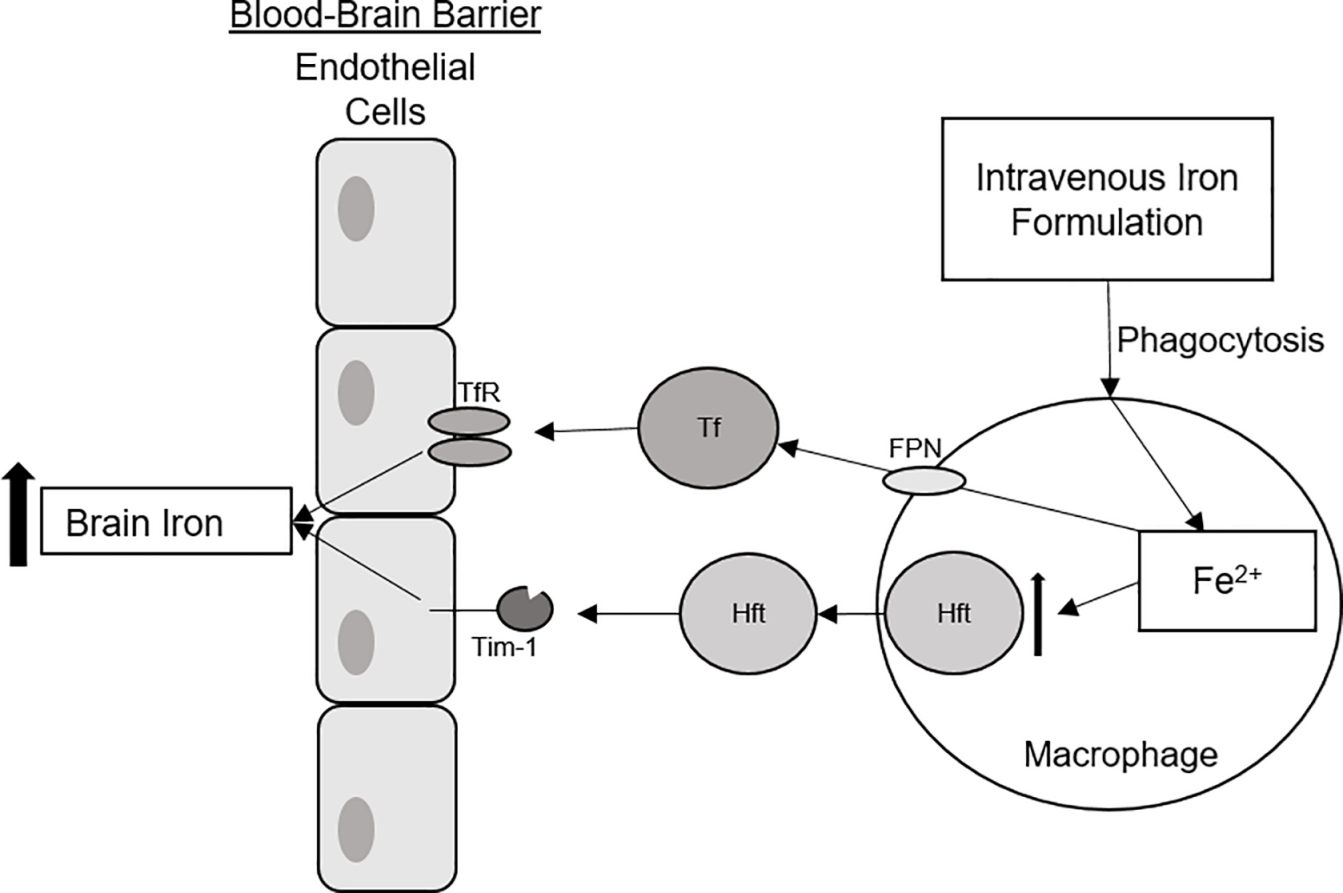
Schematic representation of hypothesized route of IV iron compound after administration. After the IV iron formulations are administered, they are may be initially picked up by macrophages. The macrophages proceed to metabolize the IV iron formulations, storing the excess iron in H-ferritin (Hft). Additionally, free iron may be exported from the macrophage via ferroportin (FPN) where it may be picked up by circulating apo-transferrin or ferritin. Transferrin (Tf) or Hft can bind to transferrin receptor (TfR) or the T-cell immunoglobulin and mucin domain 1 (Tim-1) receptor [42], and then provide iron across the BBB as demonstrated in this study.

Given that the pharmaceutical iron formulations did not load the endothelial cells with iron begs the question of how the iron in these formulations becomes bioavailable. It is generally accepted that the reticular endothelial cell system may be the first cells to accumulate iron from the iron formulations. In this paradigm, macrophages are a key component of this system and the innate immune response, serving to phagocytose foreign antigens, such as circulating drugs [37,38]. We have reported that peripheral macrophages take up the iron formulations in a cell culture model [34]. Additionally, after internalizing the iron formulations, macrophages increase release of Hft and exported free iron through ferroportin, the only known iron exporter [34]. The iron released through ferroportin would be available to bind to Tf. Indeed, when macrophages are incubated with apo-Tf in the media, they increase their iron release [39] similar to what we have reported and demonstrated herein for endothelial cells. Hepcidin, an iron regulatory hormone released by hepatocytes, was shown to block ferroportin release of iron [40]. Thus, we posit that macrophages could represent a key intermediary for making iron from the pharmaceutical formulations available bound to Hft or Tf for regulated (receptor mediated) uptake into the brain and other organs (Fig 3). It should be noted that macrophages also are key players in the iron-withholding defense mechanism during inflammation; a process mediated by hepcidin [40,41].

Pharmaceutical iron formulations have been tested clinically for treatment of neurological disorders such as Restless Legs syndrome (RLS) and have also been used as a potential contrast agent in imaging brain tumors [43,44]. Our data would suggest that for brain tumor imaging the iron compounds can only penetrate through areas where the BBB is compromised similar to the gadolinium based compounds and then accumulate in macrophages within the tumor [45,46]. In a study to determine if an IV iron compound (Monofer, iron isomaltoside-1000) can enter the brain, Unger et al. demonstrated regional correction of iron deficiency in the brain in a rodent model following a tail vein injection of iron isomaltoside-1000 [47]. This correction was observed without producing iron overload in any of the brain regions whose iron status was unchanged by the iron deficiency. Using microdialysis to detect iron in the brain, they reported a transient increase in iron uptake and that the iron was not Tf-bound. Although we did not include iron isomaltoside-1000 in our study, the regional uptake of iron observed by Unger et al. is in agreement with our findings that the pharmaceutical formulations do not cross the BBB. If iron isomaltoside-1000 natively passed through the BBB, systemic administration of iron isomaltoside-1000 it should have produced whole brain iron uptake, but only regional uptake was observed. Thus, the compound that was delivered via tail vein, may have first passed through the macrophage system as we have suggested (Fig 3).

Overall, this study serves to guide clinical treatment plans using pharmaceutical-grade intravenous iron formulations. Because the data presented herein suggest that iron formulations themselves do not directly cross the BBB, concerns that brain iron overload could result from IV iron treatments may be mitigated; albeit within the caveats associated with extrapolation from in vitro models. For treatments that are designed to alleviate symptoms of neurological disorders such as RLS, our data suggest that iron made available to the brain may first require processing in cells such as macrophages and then is subject to processes in place to regulate brain iron uptake.
